# Successful surgical repair of a sternum cleft using composite mesh: A case report and new technical note

**DOI:** 10.7196/AJTCCM.2021.v27i2.103

**Published:** 2021-06-23

**Authors:** N N M Razafimanjato, G O Tsiambanizafy, T D N Ravelomihary, H J L Rakotovao, F A Hunald

**Affiliations:** 1 Division of Thoracic Surgery, Department of Surgery, Faculty of Medicine, University of Antananarivo and Teaching Hospital of Joseph Ravoahangy Andrianavalona, Antananarivo, Madagascar; 2 Division of Pediatrics Surgery, Department of Surgery, Faculty of Medicine, University of Antananarivo and Teaching Hospital of Joseph Ravoahangy Andrianavalona, Antananarivo, Madagascar

**Keywords:** bifid sternum, chest wall deformity, congenital abnormality, laparoschisis; pentalogy of Cantrell syndrome, sternal clef

## Abstract

Sternal clefts are infrequent congenital malformations, particularly in their complete presentation. There are less than 100 descriptions of these defects published in the literature worldwide. We report a clinical case of lower sternal cleft associated with congenital laparoschisis in a 2-year-old boy. Surgery was performed because of recurrent pneumopathy and the risk of cardiorespiratory decompensation in the midterm. A semi-resorbable prosthesis was used for sternal closure. We have not observed any complications with this sternal closure system in our patient. This approach is easy, safe, effective and not harmful to a child’s growth.

## Background

Sternal clefts are a rare idiopathic chest wall deformity caused by a defect in the sternum’s congenital fusion process that can be complete or partial.^[1–2]^ In the literature, some sternal malformations are said to be major (compromising health) and non-viable (incompatible with survival), and they are associated with ‘ectopia cordis’ observed as part of a malformities named pentalogy of Cantrell.^[2]^ However, bifid sternum is usually not associated with large structural cardiac abnormalities but may be associated with trophic skin patch.^[2]^ Usually asymptomatic in the neonatal period (except for a paradoxical median thoracic swelling), it can lead to dyspnoea, cough, recurrent respiratory infections and an increased risk of traumatic injury to the heart, lungs and major vessels if it is not treated. Our patient presented with an inferior sternal cleft and upper median laparoschisis. The surgical operation consisted of the primary closure of the defect. Herein, we report a 2-year-old boy with bifid sternum beyond the neonatal period who underwent early successful primary closure using semi-resorbable composite mesh with a multidisciplinary surgical approach between anaesthesiologist, thoracic and paediatric surgeon in our centre.

## Case

**Fig. 1:**
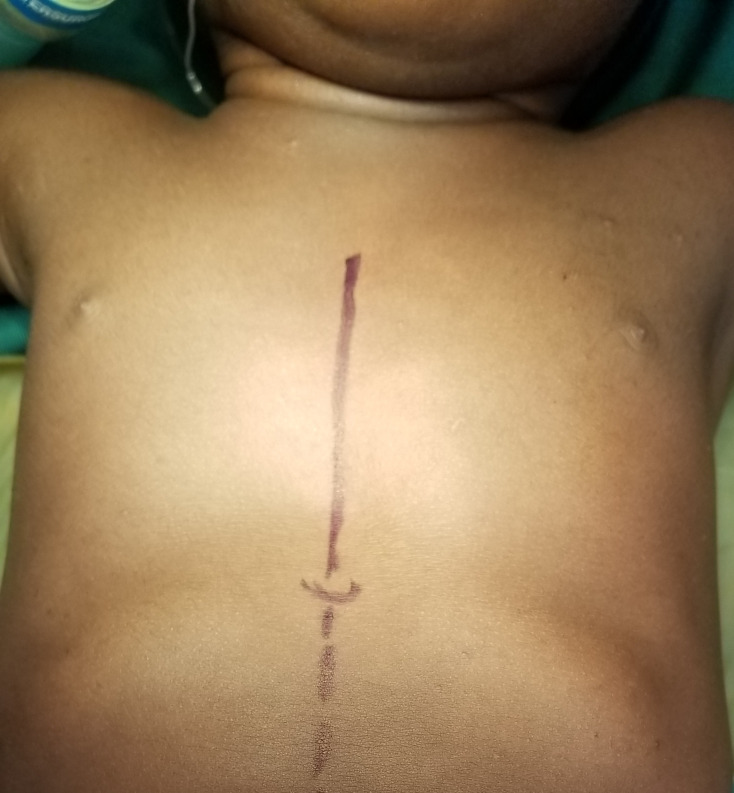
Demonstration of the paradoxical movement of the lower chest wall

A 2-year-old boy with recurrent respiratory infection and an anterior chest wall swelling was referred by the paediatrician. The child was delivered via normal vaginal birth at 36 weeks of amenorrhea, eutrophic with no mention of recurrent urinary tract infection in the mother during the pregnancy period. The obstetrical ultrasounds were uneventful. Interrogation did not reveal any parental consanguinity, no height and weight deficit and vaccination status was up to date. The parents did not report cyanosis, dyspnoea, or repeated respiratory infections in the neonatal period. On clinical examination, the child presented with thoracoabdominal balanced breathing without respiratory difficulty. Observation and palpation diagnosed a sternal cleft. Physical examination revealed an enlargement of the caudal portion of the sternum and a laparoschisis responsible for a xypho-umbilical midline eventration that worsened with crying episodes (Fig. 1).

**Fig. 2:**
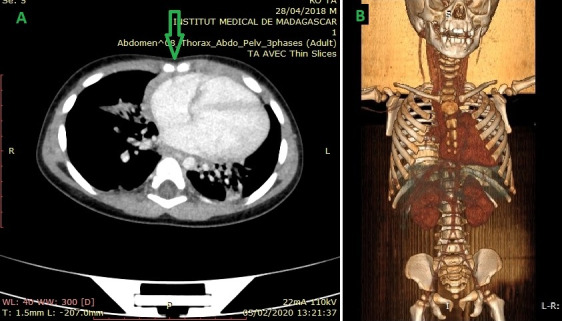
Chest axial computed tomography (CT) scan (A) demonstrates lack of fusion of the sternal margins, and (B) 3D reconstruction of the CT scan of the chest showing the edges of the lateral sternal bards.

The rest of the somatic examination was without abnormality including no associated visible malformation and a normal cardiac auscultation. The cardiac ultrasound-Doppler showed a ‘situs solitus’ without any notable cardiovascular abnormality. The chest computed tomography (CT) scan in axial sections with 3D parietal reconstruction showed the presence of small, rounded, hypodense and discontinuous formations corresponding to the ossification points in the upper part of the sternal edges and a sternal diastasis in the lower part (Fig. 2). The rest of the biological investigations revealed no abnormalities and the child’s karyotype analysis had not been investigated due to the limited financial status of the family. We made a diagnosis of lower bifid sternum associated with congenital laparoschisis in a 2-year-old male child who was completely asymptomatic and without any other associated visceral malformation.

### Technical note

**Fig. 3:**
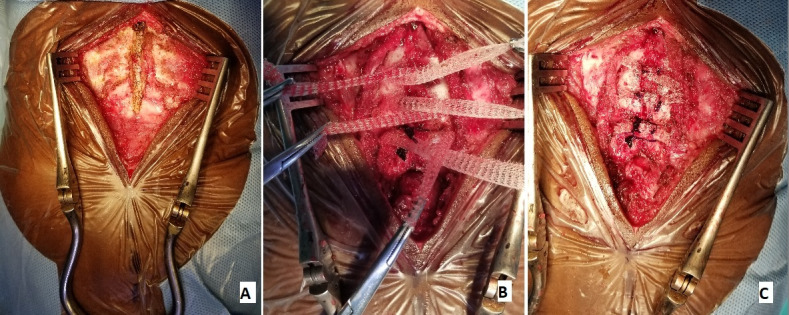
Intraoperative view of the reconstruction steps. Dissection of the anterioperiostem of the sternal bars which will be pulled medially and posteriorly (A), (B) composite mesh used to reinforce the sternum, and (C) view of the completed repair.

Primary closure of the defect was necessary given the risks of recurrent pneumopathy and long-ter m c ardio-respirator y decompensation. The operative procedure consisted of two operative steps, bone and muscular rapprochement with a composite mesh. It was performed on the patient in a supine position under general anaesthesia with ventilation via a classic orotracheal intubation. Prior to the incision, the correct anatomical landmarks (jugulum and xiphoid) had to be identified. We then undertook a median skin incision and a subcutaneous cauterisation from the fossa jugularis to the xyphoidis, followed by a layer-by-layer dissection down to the sternum to reach the sternal cleft. The sternal bars were dissected free and isolated under the periosteum from the insertion of pectoralis major muscles anteriorly, and endothoracic fascia posteriorly (Fig. 3A). The inferior sternal portion was resected as an inverted V-shape. The pre-cut prosthesis (Parietex) was inserted behind the sternum and then unrolled. Each of the tips of the prosthesis were passed through the intercostal spaces (Fig. 3B), joined together and fixed with interrupted sutures without traction on the anterior surface body of the sternum (Fig. 3C).

**Fig. 4:**
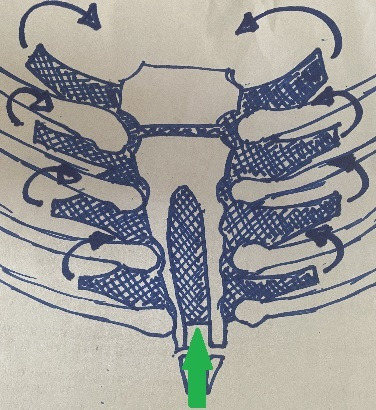
Closing of the gap between the two sternal bars behind the sternum by the prosthesis (green arrow)

The prosthesis compensated and bridged the gap between the two sternal bars (Fig. 4). In a second operative step, the diastasis of the rectus abdominis muscles was reduced by separate stitches of PDS II (poly-pdioxanone) 3/0 and the pectoral muscles were brought together with VICRYL 3/0. Retrosternal drainage was placed and the subcutaneous plane was sutured with VICRYL 4/0. There were no postoperative complications. The patient tolerated the sternal closure well without any hemodynamic instability or change in the ventilator parameters. The postoperative follow-up was uneventful. The drain was removed on day 4 post operation and the patient was discharged 7 days after repair. The result was satisfactory in the medium and long term with good cicatrisation and adequate positioning of the sternal bars giving a good aesthetic appearance. The patient is now 4 years old and in excellent condition.

## Discussion

The sternal fissure is a defect in the fusion of the sternum at an early stage of development of the fetus.^[3]^ It is a rare malformation, particularly in the form of an isolated defect that was first reported in 1740 by London.^[3]^ The first surgical correction was reported by Lannelongue in 1888, but the first successful repair was published by Burton in 1947.^[1]^ Multiple embryological studies of the sternum have attributed the sternal cleft to a defect in fusion of the two sternal bands from the lateral plate mesoderm, which normally occurs around the ninth week of intrauterine life in the craniocaudal direction.^[1,3-6]^ The cleft may involve the manubrium and upper sternebrae, the lower sternum saving the xiphoid process, but also the entire sternum. Therefore, sternal clefts are classified as incomplete and partial forms, which can be superior or inferior.^[4,5]^ According to Ravitch,^[7]^ classification of sternal clefts may be in the form of:

- Complete bifid sternum with or without ectopia cordis or herniation of pericardium known as Cantrell pentalogy (congenital sternal cleft, omphalocele, anterior diaphragmatic hernia, ectopia cordis, and a variety of intracardiac defects).
- Fusion at only upper or lower parts of sternal plates (isolated sternal defects).
- Incomplete fusion giving rise to a hole in the sternum.

We observed an association between the partial sternal cleft combined with a median xypho-umbilical laparoschisis. It should be noted that this association is infrequent. Only two other similar cases, to our knowledge, have been reported in the literature.^[8,9]^ The most common defect is the partial superior form, which affects the upper sternum and the manubrium with normal fusion of the lower part.^[5,6]^ Inferior defects are extremely rare and usually associated with complex syndromes like Cantrell pentalogy.^[4,5]^ None of this pentalogy was found in our patient. The aetiopathology of sternal clefts remains unclear. Haque^[10]^ identified familial cases in Saudi Arabia suggesting an autosomal recessive pathology. Chest wall development studies have suggested the implication of the *Hoxb-4* gene and have been inconclusively associated it with defects in mice.^[11]^ Other experiments have demonstrated that perturbations of GSK-3β (glycogen synthase kinase-3β) resulted in alterations of the para-axial mesoderm.^[12]^ Other hypotheses have been suggested, such as abuse of alcohol consumption, nutritional deficiencies and hypovitaminosis (methylcobalamin, riboflavin) during pregnancy but have not been proven.^[3,8]^ There is no reported justification for the predominantly female prevalence of the condition.^[3–5]^ However, studies by Gorlin et al. ^[13]^ and Heron *et al*. ^[14]^ showed a predominance of females when the sternal cleft is associated with a bandlike scar from the umbilicus and diastasis recti.

A bifid or sternum cleft is a rare congenital anomaly generally diagnosed as asymptomatic at birth.^[2,4-5]^ Prenatal diagnosis of isolated sternal cleft is very difficult.^[4]^ However, Yuksel *et al*.,^[15]^ diagnosed an intrauterine sternal cleft with the help of ultrasonography. In the neonatal period, the diagnosis of sternal cleft is easily done by inspection and palpation.^[6]^ Imaging based on conventional radiology, multidetector CT and magnetic resonance show the verticalisation of the clavicles, dehiscence of sternal bars, which is more important when the patient is seen at a later stage, and associated with malformations. CT is considered the best technique for studying sternal anatomy, offering the possibility to perform 3D evaluation, which can help the surgeon in surgical repair of the sternal cleft.^[5–6]^

Historically, Lannelongue^[1]^ carried out the first surgical repair of a complete sternal cleft in 1988. Burton^[8]^ published two cases of successful surgical repair by implanting a cartilage graft in the defect in the bone in 1947. Subsequently, Maier and Bortone^[16]^ carried out direct closure of two hemi-sternums in infants who were 6 weeks old and this technique has become the reference.^[8]^ Whether symptomatic or not, the sternal cleft requires surgical correction in the new born, when the flexibility of the chest wall is maximal and compression of underlying structures is minimal to restore bony protection to the mediastinal structures, establish normal intrathoracic pressure relationships, improve respiratory dynamics and for aesthetic reasons.^[2,4,6]^ In the literature, repairs performed after the age of 3 months have always required more supportive postoperative care, with a higher incidence of cardiac complication. ^[5,8]^ Beyond one year, autologous grafts (costal cartilage, parietal skull, tibial periosteum) or prosthetic materials such as Marlex mesh, Teflon mesh, silicone elastomer and acrylic are required.^[5–6]^ Moreover, studies have suggested that it is always preferable to use autogenous tissue for sternal cleft repair and to avoid the use of prosthetic materials, recognising the risk of infection and the negative impact of inert material on the patient’s development.^[1,4,8]^

Despite the risk of infection described in the literature, the use of prosthesis in sternal cleft repair has an advantage in preventing an increase in intrathoracic pressure such as explained in our technical note.^[17]^ In addition, this surgical approach avoids major surgical dissection and the use of autogenous or bone bank rib reconstruction, which presents a potential risk of aseptic or septic necrosis of the bone.^[18]^ What sets Parietex apart from other prosthetic material is the density it can achieve from relatively little pressure. Most other prosthetics take thousands of pounds of pressure to squeeze them into soft shapes. The other benefits of using this type of prosthesis are its flexibility and conformity to the anatomy once implanted. The resorbable hydrophilic film promotes rapid parietal integration to provide a temporary barrier and minimise visceral attachment to mesh.^[19]^ The nonabsorbable component provides stability and bridge the gap between the two sternal bars while avoiding direct approximation of both sternal halves and to prevent increasing intrathoracic pressure. It allows fast and complete tissue in-growth on one side for efficient reinforcement. A study by Casha *et al*.^[20]^ evaluated and quantified the rigidity of sternotomy fixation using a mechanical model through which six different fixation techniques were tested: figure of 8, straight, Ethibond, repair, Sternaband and multitwist, and showed that Sternaband with its flat ribbon shape, similar to the cutting of the prosthesis in our technique, is more resistant to cutting than wires. The force imparted by the lateral part of the closure can help bring direct approximation of both sternal halves, providing stability. To the best of our knowledge, our case is the first reparation of sternal cleft using composite mesh and since this defect is so rare, we don’t have a vast experience on this subject. However, this surgical approach with mesh prosthesis can be indicated in many types of sternal clefts (superior sternal cleft, subtotal sternal cleft, total sternal cleft, inferior sternal cleft and median sternal cleft) to guarantee favourable long term results with reference to the stature growth, reproducibility, and adaptsbility to all surgical centres that train in general surgery.

## Conclusion

The neonatal period is the best period for surgical correction of sternal clefts due to the elasticity of the sternum and minimal compression of the underlying structures. All infants with recurrent pulmonary infection should be referred to surgeons for an early investigation to detect malformation of either the lungs or the chest wall to avoid delayed surgical repair. A considerable variety of sternal cleft repair procedures have been reported in the literature. However, our surgical procedure is safe and relatively easy, preventing any impact on the child’s stature and postural development.
